# Changes in psychiatric disorder incidence patterns during the COVID-19 pandemic in Korea: a study using the nationwide universal health insurance data

**DOI:** 10.1186/s12888-024-06325-7

**Published:** 2024-12-05

**Authors:** Changwoo Han, Juho Choi, Hoyeon Jang, Hwa-Young Lee, Tarik Benmarhnia, Juhwan Oh

**Affiliations:** 1 Department of Preventive Medicine, Chungnam National University College of Medicine, 266, Munhwa-Ro, Jung-Gu, Daejeon, 35015 Republic of Korea; 2 Scripps Institution of Oceanography, University of California, San Diego, CA 92037 USA; 3 Department of Preventive Medicine, College of Medicine, Seoul National University, Seoul, Republic of Korea; 4 Department of Big Data Research and Development, National Health Insurance Service, Wonju, Republic of Korea; 5 Graduate School of Public Health and Healthcare Management, The Catholic University, Seoul, Republic of Korea; 6 Université de Rennes, Inserm, EHESP, Irset (Institut de Recherche en Santé, Environnement Et Travail) - UMR_S 1085, 35000 Rennes, France; 7 Seoul National University College of Medicine, Seoul, Republic of Korea

**Keywords:** COVID-19, Excess disease burden, Psychological disease, Interrupted time series analysis

## Abstract

**Background:**

Few studies have evaluated the incidence of various psychiatric disorders during the coronavirus disease (COVID-19) pandemic using hospital visit data for the entire population of a nation. We used Korea’s universal compulsory health insurance data to conduct a descriptive analysis to evaluate changes in psychiatric disorder incidence during the COVID-19 pandemic.

**Methods:**

Hospital visit data related to psychiatric disorders were retrieved from the National Health Insurance Database. First-ever diagnosis for specific psychiatric disorders for each Korean was identified (from January 2015 to February 2023) and monthly age-standardized incidence rates were calculated. An interrupted time-series analysis was used to estimate counterfactual disease incidence rates and observed and counterfactual rates were compared using difference-in-difference framework.

**Results:**

Compared to pre-pandemic period, there was a decrease in the incidence [percentage changes in monthly rate (95% confidence intervals)] of organic mental disorders [-14.6% (-18.4, -10.9)] and psychoactive substance related disorders [-12.9% (-17.5, -8.3)] during the pandemic. However, anxiety disorders [8.8% (3.1, 14.6)], behavioral syndromes associated with physiological disturbances [8.1% (4.2, 11.9)], mental retardation [8.6% (3.0, 14.2)], psychological developmental disorders [19.6% (11.5, 27.7)], childhood- and adolescent-onset behavioral and emotional disorders [45.1% (28.4, 61.8)], and unspecified mental diseases [51.8% (39.8, 63.8)] increased.

**Conclusions:**

Psychological disease incidence patterns changed substantially during the pandemic in South Korea. Various pandemic-related stressors, such as disrupted lifestyles and hospital accessibility, may have influenced these changes. The causes and public health consequences of these changes require further evaluation.

**Supplementary Information:**

The online version contains supplementary material available at 10.1186/s12888-024-06325-7.

## Background

By end of August 2023, 34,572,554 coronavirus disease (COVID-19) cases and 35,605 deaths were reported in South Korea [1]. Apart from infection-related burden, deterioration in the metal health may have occurred due to the factors such as fear of COVID-19 infection or vaccination, changes in livelihood and socioeconomic conditions, reduced social interactions, and stringent government policies [2–5]. Several countries reported changes in suicidal patterns and an increase in psychiatric problems during the pandemic as well [3, 6, 7].

Identifying the mental health effect following unprecedented events like the COVID-19 pandemic is an important public health task [8, 9]. Because the each response from different sovereignty varied across countries, it is uncertain whether findings from one country can be directly applied to others [10, 11]. Therefore, it is necessary to quantitatively assess how pandemic events affect various psychiatric disorders in each country.

Studies in South Korea have reported an increase in mental stress since 2020 [12–16]. Surveys of nationally-representative adolescent populations have shown increasing trends in stress levels [17]. However, most Korean studies have involved a limited number of participants, utilized cross-sectional study designs focused on specific periods of pandemic, and evaluated mental health using self-reported questionnaires [5, 12–16]. Therefore, research based on objective data with physician diagnoses for a comprehensive set of psychiatric disorders throughout the entire pandemic period is still limited.

An analysis of excess disease incidence may help estimate the overall burden of the COVID-19 pandemic on mental health [18]. However, this study is only feasible in regions where the entire medical service utilization of each study subject can be assessed [18]. South Korea’s distinctive health insurance system records lifetime hospital utilization information for the entire population [19]. Consequently, South Korea’s health insurance data can serve as a relevant resource for investigating the new onset of diseases during the pandemic, diagnosed by trained physicians. However, previous studies using insurance or hospital data in Korea have focused on the total number of hospital visits related to psychiatric disorders and did not consider the influence of long-term trends and seasonal variations of hospital visits, and age-demographic shifts during the study period [20–22].

Therefore, based on the assumption that the pandemic may have affected wide range of mental health condition of the population, this study aims to conduct a descriptive analysis to quantify the impact of COVID-19 on various psychiatric disorders by using nationwide health insurance data. By focusing on first-ever diagnosis of specific psychiatric disorders of each Korean, the changes in disease incidence patterns during the pandemic were estimated.

## Material and methods

### Health insurance data

South Korea operates a compulsory health insurance system covering the entire Korean population administered by the National Health Insurance Service (NHIS) [23]. The NHIS has established a data warehouse called the National Health Information Database (NHID) that includes information on healthcare utilization, health screening results, and demographic variables for the entire Korean population [19]. Information on hospital visits, including dates, diagnostic codes, prescription records, and demographic data, such as birth year, sex, residential address, and date of death, are also available [19, 23].

One study that compared the yearly eligible NHID insurance policyholder data with mid-year population data generated from official statistics provided by Statistics Korea for the period of 2004–2015 found that 98–100% of the Korean population is included in the NHID [23]. Therefore, previous studies have utilized the NHID to evaluate changes in healthcare utilization patterns or the onset of new diseases following events such as the COVID-19 pandemic or natural disasters [24–26]. The annual population of Korea in 2020 (*n* = 51,840,130) and for each year during the study period (2013 to 2023) is presented in Table S1 (see Additional File 1).

In May 2022, the NHIS and Korea Centers for Disease Control and Prevention Agency initiated a public–private joint research initiative to facilitate evidence-based decision-making regarding COVID-19-related public health issues. Our study was conducted as part of this initiative and utilized NHID.

### Disease incidence

Hospital visit data for the following major psychiatric disorder categories were retrieved from the NHID using International Classification of Diseases (ICD-10) codes of primary diagnosis: organic, including symptomatic, mental disorder (F00-F09); mental and behavioral disorders due to psychoactive substance use (F10-F19); schizophrenia, schizotypal, and delusional disorders (F20-F29); mood disorders (F30-F39); neurotic, stress-related, and somatoform disorders (F40-F48); behavioral syndromes associated with physiological disturbances and physical factors (F50-F59); adult personality and behavioral disorders (F60-F69); mental retardation (F70-F79); psychological developmental disorders (F80-F89); behavioral and emotional disorders usually with childhood and adolescent onset (F90-F98); and mental disorder not otherwise specified (NOS) (F99).

The term ‘disease incidence’ was used to define new onsets of disease in each individual that did not emerge for at least two years. From the National Health Insurance Database, we initially extracted all hospital visits (which allows multiple visits from single individual) of entire Korean diagnosed with each disease category from January 2013 to February 2023. Subsequently, for each individual and disease category, we identified the date of first ever hospital visit (does not allow multiple visits and unique for each individual) and defining them as timing of disease development. Given that our study period spans from January 2015 to February 2023, if an individual's first visit with 'A' disease diagnosis occurred between January 2015 and February 2023, it confirms that the individual was not diagnosed with 'disease A' for at least two years. For instance, if a person's initial hospital visit for 'other diseases of the mood disorder (F30-F39)' was observed on March 2020, this indicates that the person had not visited the hospital for that specific disease from January 2013 to March 2020. In sensitivity analyses, we applied different study periods (January 2016 to February 2023, January 2017 to February 2023) to provide alternative definitions of disease incidence (identifying new occurrences of disease in each individual that had not emerged for at least three or four years, respectively). Based on 5-year age groups and sex, monthly age-standardized incidence rates were calculated for temporal comparisons to determine the age-related structural change during the study period [4, 27].

### Statistical analysis

To evaluate the changes in disease incidence patterns for each psychiatric disorder categories during the pandemic, we compared the observed age-standardized disease incidence rates to that of the counterfactual incidence rates (i.e., the rates that might have been observed without the pandemic) estimated using an interrupted time-series model.

The following interrupted time series model was fitted for each disease category:

1 $${\mu }_{t}={\beta }_{0}+{\beta }_{1}{I}_{t}+{\beta }_{2}{T}_{t}+{\beta }_{3}{I}_{t}P+{\beta }_{4}ns\left(M, df=8\right)+\varepsilon$$

In Eq. 1, ${\mu }_{t}$ represents the age-standardized incidence rate in year-month t and is assumed to follow normal distribution. ${I}_{t}$ is a categorical variable representing the pandemic (${I}_{t}$ = 0: before the pandemic, January 2015–February 2020; ${I}_{t}$ = 1: during the pandemic, March 2020–February 2023), ${T}_{t}$ represents the time elapsed in months since the study started (January 2015), and *P* represents the time in months since the pandemic started (set as March 2020 based on previous studies [4]). We applied eight degrees of freedom for the calendar month (M) with a natural spline term, which was selected based on the lowest Akaike Information Criterion values in the model [28]. After fitting the interrupted time-series model, the "predict” function in R software was used to estimate the counterfactual incidence rate (c_t_: hypothetical incidence rates which might be observed in the absence of the COVID-19 pandemic) for the entire study period (January 2015–February 2023).

We then calculated [29] the difference between the observed age-standardized incidence rates ($\text{E}\left({\text{Y}}_{t}\right)={\mu }_{t}$ when *R* = 1) and estimated counterfactual age-standardized rates ($\text{E}\left({\text{Y}}_{t}\right)=$ c_t_ when *R* = 0) during the pandemic (March 2020-February 2023) using the following difference-in-difference equation:

2 $$\text{E}\left({\text{Y}}_{t}\right)= {\beta }_{0}+{\upbeta }_{1}R+{\upbeta }_{2}{I}_{t}+{\upbeta }_{3}R*{I}_{t}+\varepsilon$$

In Eq. 2, ${\beta }_{3}$ was used to estimate the average change in disease incidence, which shows the average difference between the observed and estimated monthly age-standardized psychiatric disorder incidence patterns during the pandemic.

To confirm the validity of the difference-in-difference analysis, we first compared the parallel trends between observed and estimated incidence rates before the pandemic period (Figures S1 and S2, see Additional file 1). Second, we selected time points 2 years before the start of the pandemic in South Korea (March 2018) and compared the differences between observed and estimated age-standardized incidence rates for the hypothetical pandemic period (March 2018 to February 2020) as part of a negative control analysis. No significant differences were found between observed and estimated incidence rates before the pandemic (Table S2, see Additional file 1).

Stratification and sensitivity analyses were conducted. First, a sex-stratified analysis was conducted based on previous studies suggesting that sex can modify the impact of the COVID-19 pandemic on mental health [5, 30]. Differences between estimates were addressed using Cochran Q statistics [31]. Second, we divided the study period into each year (March 2020–February 2021; March 2021–February 2022; March 2022–February 2023) after the start of the pandemic in Korea and evaluated whether changes in disease incidence pattern differed as the pandemic continued. Third, we applied different definitions of study period by limiting the pre-pandemic period as follows: January 2016–February 2020 and January 2017–February 2020 to model counterfactual age-standardized rate (c_t_). Analyses were performed using SAS (version 9.4; SAS Institute Inc., Cary, NC, USA) and R statistical software (version 4.2.2; R Foundation for Statistical Computing, Vienna, Austria).

## Results

**Fig. 1:**
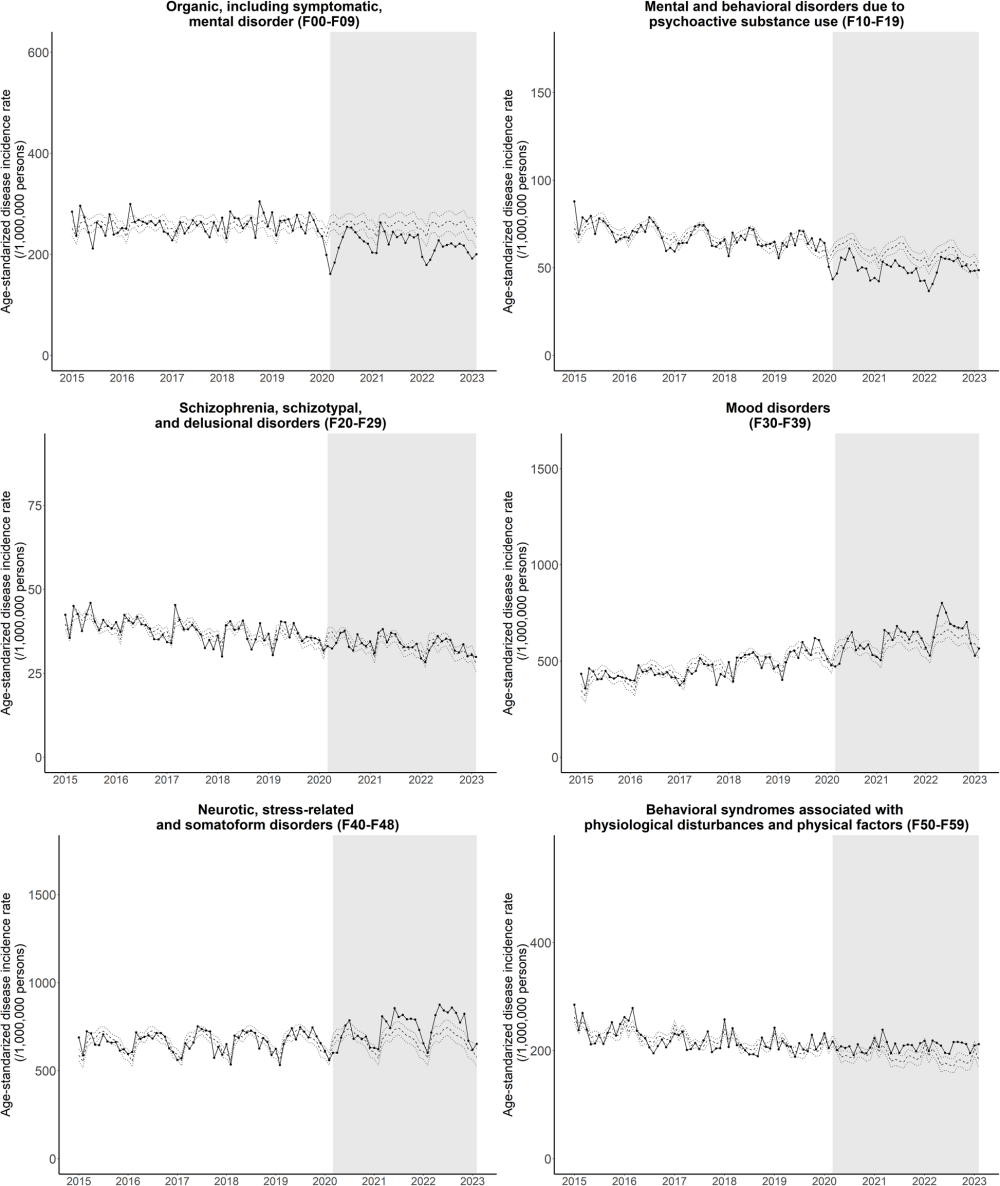
Age-standardized monthly disease incidence rates of psychiatric disease (F00 to F59) from January 2015 to February 2023. Middle dotted line represents estimated rates based on the pre-COVID19 pandemic disease incidence trend and solid line represents observed values. Upper and lower dotted lines represent 95% confidence intervals of the estimated rates

**Fig. 2:**
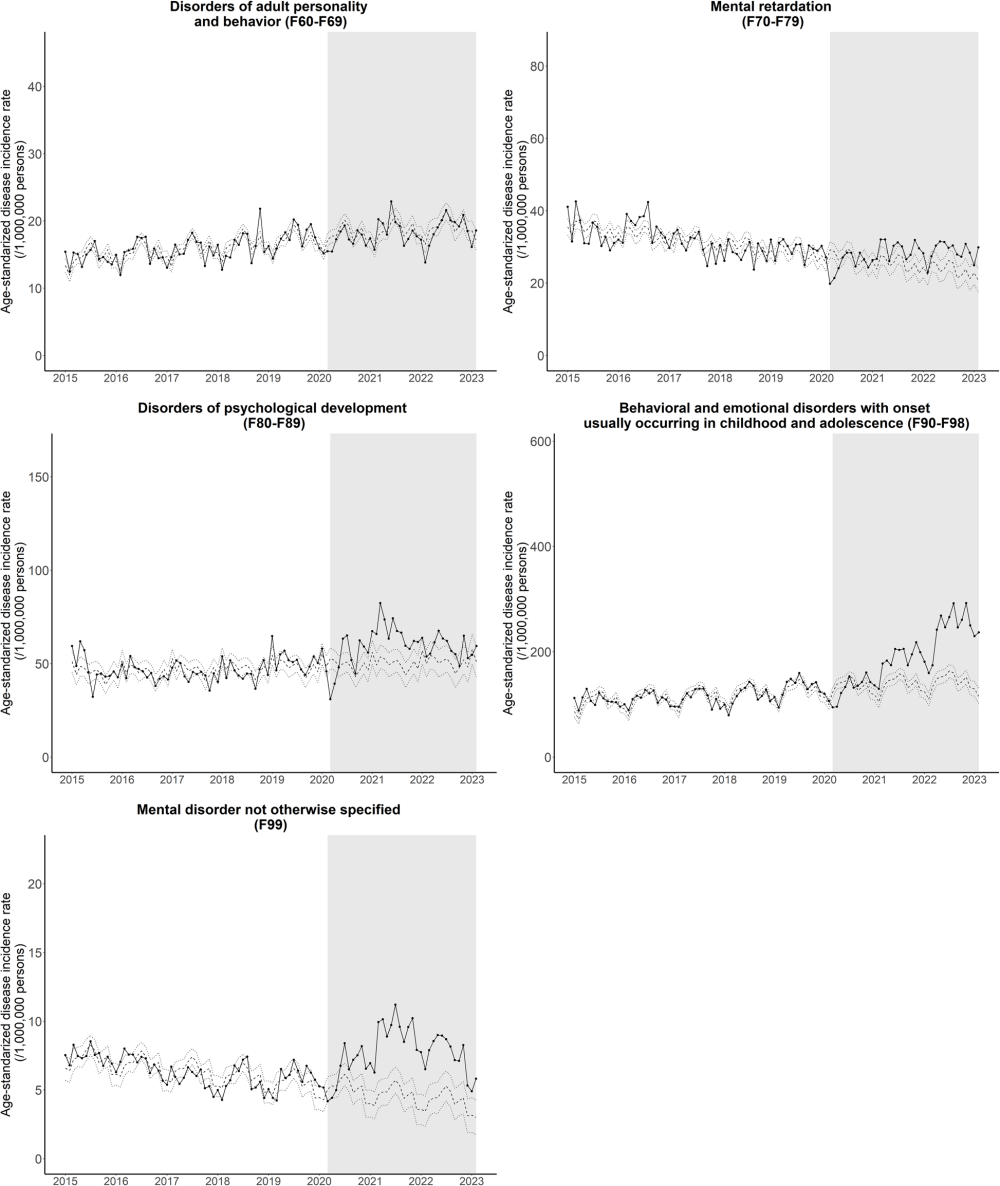
Age-standardized monthly disease incidence rates of psychiatric disease (F60 to F99) from January 2015 to February 2023. Middle dotted line represents estimated rates based on the pre-COVID19 pandemic disease incidence trend and solid line represents observed values. Upper and lower dotted lines represent 95% confidence intervals of the estimated rates

Table 1 and Figs. 1 and 2 show the changes in monthly age-standardized psychiatric disorder incidence patterns during the pandemic. The patterns of observed and estimated counterfactual incidence rates were compatible (showing parallel trends) before the pandemic (Figs. 1 and 2, Figures S1 and S2, see Additional file 1). We observed heterogenous patterns across disease categories during the pandemic.

**Table 1:** Changes in monthly age-standardized disease-specific incidence rate during the COVID-19 pandemic

Disease categories (ICD-10 code)	Monthly disease incidence count (n)		Monthly disease incidence rate (/1,000,000)		Changes in monthly disease incidence rate^c^ (/1,000,000)	Percent change compared to prepandemic^d^(%)
	Before pandemic^a^	During pandemic^b^	Before pandemic^a^	During pandemic^b^		
Organic, including symptomatic, mental disorder (F00-F09)	23317	23631	258	220	-37.78 (-47.52, -28.03)^e^	-14.6 (-18.4, -10.9)^e^
Mental and behavioral disorders due to psychoactive substance use (F10-F19)	4317	3067	68	50	-8.76 (-11.89, -5.63)^e^	-12.9 (-17.5, -8.3)^e^
Schizophrenia, schizotypal and delusional disorders (F20-F29)	2358	2062	38	34	0.11 (-1.49, 1.71)	0.3 (-3.9, 4.5)
Mood disorders (F30-F39)	28619	33922	468	617	22.42 (-12.49, 57.33)	4.8 (-2.7, 12.2)
Neurotic, stress-related and somatoform disorders (F40-F48)	41984	43246	664	737	58.51 (20.39, 96.63)^e^	8.8 (3.1, 14.6)^e^
Behavioral syndromes associated with physiological disturbances and physical factors (F50-F59)	15599	15153	221	208	17.82 (9.26, 26.38)^e^	8.1 (4.2, 11.9)^e^
Disorders of adult personality and behavior (F60-F69)	729	790	16	18	-0.43 (-1.41, 0.55)	-2.7 (-8.8, 3.4)
Mental retardation (F70-F79)	1211	939	32	28	2.76 (0.96, 4.56)^e^	8.6 (3.0, 14.2)^e)^
Disorders of psychological development (F80-F89)	1277	1317	47	60	9.21 (5.40, 13.01)^e^	19.6 (11.5, 27.7)^e^
Behavioral and emotional disorders with onset usually occurring in childhood and adolescence (F90-F98)	3494	5763	116	191	52.28 (32.93, 71.64)^e^	45.1 (28.4, 61.8)^e^
Mental disorder NOS (F99)	324	375	6	8	3.11 (2.39, 3.83)^e^	51.8 (39.8, 63.8)^e^

^a^Before pandemic: 2015.01—2020.02

^b^During pandemic: 2020.03—2023.02

^c^Comparison between observed and predicted counterfactual incidence rate

^d^Changes in monthly disease incidence rate/monthly disease incidence rate before pandemic

^e^*p*-value below 0.05

Compared to pre-pandemic rates, the incidence [percentage changes in monthly age-standardized rate per 1,000,000 persons (95% confidence intervals)] of organic, including symptomatic, mental disorders [-14.6% (-18.4, -10.9)] and psychoactive substance use-related mental and behavioral disorders [-12.9% (-17.5, -8.3)] decreased during the pandemic; however, the incidence of schizophrenia, schizotypal, and delusional disorders [0.3% (-3.9, 4.5)] and mood disorders [4.8% (-2.7, 12.2)] did not change (Table 1 and Fig. 1).

There was an increase in the incidence of neurotic, stress-related, and somatoform disorders [8.8% (3.1, 14.6)] and behavioral syndromes associated with physiological disturbances and physical factors [8.1% (4.2, 11.9)] (Table 1 and Fig. 1), as well as diseases such as mental retardation [8.6% (3.0, 14.2)], psychological developmental disorders [19.6% (11.5, 27.7)], behavioral and emotional disorders usually with childhood and adolescent onset [45.1% (28.4, 61.8)], and mental disorder NOS [51.8% (39.8, 63.8)] (Table 1 and Fig. 2).

Tables 2 and S3 present the results of the sex-stratified analysis. Significant sex-based differences were observed in the incidence of organic, including symptomatic, mental disorders; mood disorders; neurotic, stress-related, and somatoform disorders; and psychological developmental disorders. Although women showed greater changes in the incidence patterns of the former three diseases, men showed greater changes in the incidence patterns of psychological developmental disorders.

**Table 2:** Gender-stratified estimation of the changes in monthly age-standardized disease-specific incidence rate during the COVID-19 pandemic

Disease categories (ICD-10 code)	Monthly disease incidence count (n)		Monthly disease incidence rate (/1,000,000)		Changes in monthly disease incidence rate^c^ (/1,000,000)	Percent change compared to prepandemic^d^(%)
	Before pandemic^a^	During pandemic^b^	Before pandemic^a^	During pandemic^b^		
Men						
Organic, including symptomatic, mental disorder (F00-F09)	7905	8514	219	193	-27.22 (-35.4, -19.03)^e^	-12.4 (-16.2, -8.7)^e^
Mental and behavioral disorders due to psychoactive substance use (F10-F19)	3045	2096	92	63	-7.16 (-11.98, -2.33)^e^	-7.8 (-13.0, -2.5)^e^
Schizophrenia, schizotypal and delusional disorders (F20-F29)	1083	912	37	31	0.42 (-1.21, 2.05)	1.1 (-3.3, 5.5)
Mood disorders (F30-F39)	10732	12538	363	457	-0.07 (-24.74, 24.61)	0.0 (-6.8, 6.8)
Neurotic, stress-related and somatoform disorders (F40-F48)	16261	17118	540	602	29.99 (1.43, 58.56)^e^	5.6 (0.3, 10.8)^e^
Behavioral syndromes associated with physiological disturbances and physical factors (F50-F59)	6562	6417	195	183	12.34 (4.94, 19.73)^e)^	6.3 (2.5, 10.1)^e^
Disorders of adult personality and behavior (F60-F69)	518	561	22	25	0.05 (-1.33, 1.44)	0.2 (-6.0, 6.5)
Mental retardation (F70-F79)	763	597	39	36	3.19 (0.96, 5.43)^e^	8.2 (2.5, 13.9)^e^
Disorders of psychological development (F80-F89)	933	936	67	83	12.9 (7.48, 18.32)^e^	19.3 (11.2, 27.3)^e^
Behavioral and emotional disorders with onset usually occurring in childhood and adolescence (F90-F98)	2387	3512	156	235	52.77 (29.37, 76.17)^e^	33.8 (18.8, 48.8)^e^
Mental disorder NOS (F99)	177	188	7	8	3.15 (2.3, 4.01)^e^	45.0 (32.9, 57.3)^e^
Women						
Organic, including symptomatic, mental disorder (F00-F09)	15411	15117	289	245	-47.5 (-58.96, -36.05)^e^	-16.4 (-20.4, -12.5)^e^
Mental and behavioral disorders due to psychoactive substance use (F10-F19)	1272	970	45	36	-10.34 (-12.5, -8.17)^e^	-23.0 (-27.8, -18.2)^e^
Schizophrenia, schizotypal and delusional disorders (F20-F29)	1274	1150	39	36	-0.04 (-1.76, 1.68)	-0.1 (-4.5, 4.3)
Mood disorders (F30-F39)	17886	21384	576	785	45.13 (-1.71, 91.96)	7.8 (-0.3, 16.0)
Neurotic, stress-related and somatoform disorders (F40-F48)	25723	26128	788	878	86.77 (37.61, 135.92)^e^	11.0 (4.8, 17.2)^e^
Behavioral syndromes associated with physiological disturbances and physical factors (F50-F59)	9037	8736	249	236	23.96 (13.72, 34.21)^e^	9.6 (5.5, 13.7)^e^
Disorders of adult personality and behavior (F60-F69)	211	229	10	11	-0.87 (-1.53, -0.22)^e^	-8.7 (-15.3, -2.2)^e^
Mental retardation (F70-F79)	447	342	23	20	2.28 (0.85, 3.71)^e^	9.9 (3.7, 16.1)^e^
Disorders of psychological development (F80-F89)	343	381	26	35	5.28 (3.02, 7.55)^e^	20.3 (11.6, 29)^e^
Behavioral and emotional disorders with onset usually occurring in childhood and adolescence (F90-F98)	1107	2252	73	144	51.97 (36.22, 67.71)^e^	71.2 (49.6, 92.8)^e^
Mental disorder NOS (F99)	147	187	5	7	3.06 (2.42, 3.69)^e^	61.2 (48.4, 73.8)^e^

^a^Before pandemic (2015.01—2020.02)

^b^During pandemic (2020.03—2023.02)

^c^Comparison between observed and predicted counterfactual incidence rate

^d^Changes in monthly disease incidence rate/monthly disease incidence rate before pandemic

^e^*p*-value below 0.05

Distinct changes were observed in disease incidence patterns across the pandemic years (Table S4, see Additional file 1). Specifically, continuously increasing trends were observed for following categories: neurotic, stress-related, and somatoform disorders (percentage changes in disease incidence patterns compared to pre-pandemic, 2020: -0.5%, 2021: 12.9%, 2022: 14.1%); behavioral syndromes associated with physiological disturbances and physical factors (2020: 3.7%, 2021: 8.8%, 2022: 11.7%); mental retardation (2020: -3.9%, 2021: 8.8%, 2022: 17.5%); and childhood and adolescent behavioral and emotional disorders (2020: -1.2%, 2021: 44.4%, 2022: 92.1%). Notably, the greatest changes were observed during year 2021 in psychological developmental disorders (2020: 10.7%, 2021: 32.4%, 2022: 15.7%) and mental disorder NOS (2020: 24.8%, 2021: 76.0%, 2022: 55.2%). Sensitivity analyses with different definitions of pre-pandemic period (January 2016–February 2020 and January 2017–February 2020) produced results similar to those of the main analysis (Tables S5 and S6, see Additional file 1).

## Discussion

Using Korea’s health insurance data, we examined changes in psychiatric disorder incidence patterns throughout the pandemic to determine its influence on the nation's mental well-being. Notably, there was an observable increase in anxiety-related disorders and conditions linked to physiological disruptions during the pandemic. The incidence of diseases that manifest early in life, such as psychological developmental disorders, childhood- and adolescent-onset behavioral and emotional disorders, and mental retardation, increased during the pandemic. While the incidence rates of organic, including symptomatic, mental disorders, and disorders due to psychoactive substance use decreased, those of schizophrenia and mood disorders remained constant.

Increased social isolation and fear of COVID-19 infection, disruption of normal daily lives and primary care mental health services, financial strain due to socioeconomic change, and strict government policies may have contributed to increased external stress and psychiatric disorders during the pandemic [2, 3, 5]. In our study, we found an increased number of patients with conditions linked to physiological disruptions and mental health symptoms that did not meet the diagnostic criteria for specific diseases (mental disorders NOS). Other Korean studies have confirmed these trends by consistently reporting increased physiological and mental stress during the pandemic [12–16].

The incidence of anxiety-related disorders (F40-F48) markedly increased since 2021. During this period, Koreans experienced a rapid epidemic of omicron variants and increased mortality rates after the successful management of the pandemic in 2020 [32, 33]. Pooled studies from 15 countries reported mental health deterioration following an increase in the number of COVID-19 infections/deaths and introduction of stricter policies [2].

The incidence of childhood and adolescent behavioral and emotional disorders markedly increased. Surveys of nationally-representative adolescent samples also reported a continuously increasing trend in adolescents reporting severe stress [boys: 28.1% (2020), 32.3% (2021), 36.0% (2022); girls: 40.7% (2020), 45.6% (2021), 47.0% (2022)] during the pandemic [17]. In contrast, the prevalence of perceived health as good or very good decreased [boys: 74.3% (2020) 69.7% (2021), 68.2 (2022); girls: 64.6% (2020), 59.3% (2021), 57.8% (2022)] [34]. Unprecedented changes during the pandemic, such as school closures, social distancing, and changes in daily activities, may have negatively affected the mental health of children and adolescents [3, 6, 12, 30]. A meta-analysis of 29 studies on children and adolescents revealed a 25% and 20% increase in the rates of depression and anxiety, respectively, during the pandemic [3]. On pooling 21 repeated-measures cross-sectional or longitudinal studies, marked mental health deterioration, including an increase in the rates of depression, anxiety, loneliness, and distress, was reported [6].

We found an increase in the incidence rates of developmental disorders including disorders of speech, language, scholastic skills, motor function development, and pervasive disorders, such as autism spectrum disorder (ASD) especially during the year 2021. A child’s development occurs through social interaction, but social distancing from the beginning of the pandemic may limit their chances of interacting with others and negatively affect overall developmental processes [5, 35].

Korean kindergartens were closed during the early pandemic years, and preschool children may have spent less time with their peers than before [36]. On comparing Korean children’s leisure activities between 2020 and 2019, a decline in the time spent reading, engaging in physical activities, and playing with friends, whereas an increase in the time spent using smartphones and computers was observed [37]. Schools were closed in March 2020 and fully reopened in mid-June, but remote classes were facilitated throughout the pandemic in South Korea [36]. Even after reopening, students attended classes every other week/day, and schools were temporarily closed depending on the number of COVID-19 cases in a given class or the local region from 2020 to early 2021 [30].

Remote learning classes negatively affected the cognitive and social development of Korean children aged 10–17 [38]. Several studies worldwide have discussed the association between social isolation and worsening child development [39, 40]. The unprecedented changes that occurred during the early stages of the pandemic, including school closures, stringent social distancing measures, and disruptions to daily lives, may have had a detrimental impact on childhood development, potentially leading to an increased incidence of developmental disorders during year 2021 with a 1-year time lag. Additionally, ASD patients reported higher perceived stress levels than did those without ASD during the pandemic [41]. This stress may have led to an even more pronounced expression of typical ASD symptoms [42], potentially resulting in increased hospital visits and diagnosis of disease.

Audiovisual attention is a key mechanism of speech-production ability [43]. However, masks may negatively affect the communication process between the child and caregiver by reducing the speech signal and obscuring articulatory gestures and facial expression [44, 45]. In South Korea, mandatory mask use in public spaces introduced in November 2020 was expanded to cover all indoor areas by April 2021. While the compulsory outdoor mask mandate was gradually relaxed from May 2022 [46], the requirement to wear masks indoors remained in effect until January 2023. Therefore, widespread mask use may have negatively affected child development.

According to an annual report on disability statistics, the number of newly reported Koreans with intellectual disabilities increased by 2021 after a sharp decrease in 2020 (2019: 14,690, 2020: 13,801, 2021: 14,428) [47], coinciding with our findings. A reduction in mental illness-related hospital visits was observed in South Korea and other countries, particularly during the early pandemic [48]. This may have led to a delayed diagnosis of serious diseases, such as mental retardation, resulting in an increased disease incidence pattern since year 2021.

The incidence pattern of schizophrenia and delusional disorders was relatively stable. A study in a tertiary hospital in South Korea showed that healthcare service utilization patterns of patients with schizophrenia differed from those of patients with other mental illnesses during the pandemic [21]. The daily outpatient visit rates for anxiety and depressive disorders, but not for schizophrenia-spectrum disorders, were significantly associated with the daily number of newly confirmed COVID-19 cases. This suggests that the pandemic’s effect on hospital visits differed according to the type of mental disease [21]. A recent review article reported that patients with schizophrenia reported relatively stable mental stress, symptoms, and social well-being compared with patients with anxiety or depressive disorders during the pandemic [49].

The incidence rates of organic, including symptomatic, mental disorders markedly decreased during the pandemic. Since elderly individuals are a high-risk group for COVID-19 mortality, they are often subjected to intense social isolation [50]. During the pandemic, a notable decrease in attendance at family gatherings and social meetings and number of hospital and health center visits as well as utilization of welfare centers and senior citizen community centers was reported among elderly Koreans [51]. Heightened social isolation could lead to decreased elderly hospital visits, which may delay the diagnosis of conditions, such as dementia.

Although the use of substance increased with various mental stress accompanying pandemic [52, 53], disease incidence related to psychoactive substance use showed decreasing pattern in our study. The accessibility to illicit substances may have decreased during the period of social distancing and lockdown [53, 54], but further evaluation is needed to evaluate the exact cause of decrease. While the overall analysis did not reveal any changes in mood disorders, the sex-stratified analysis indicated a significant increase in the incidence of psychiatric disorders in women (Table S3, see Additional file 1). In addition, there was a greater increase in anxiety-related disorders among women than among men. These results were anticipated based on a global report that women were relatively more burdened with household chores and childcare responsibilities, received lower wages, and were more likely to be victims of domestic violence during the pandemic [5].

This study demonstrates that the COVID-19 pandemic disrupted the ongoing incidence patterns of diverse psychiatric disorders in Korea. Further research is needed to identify possible causes and manage these newly increased patient groups. Because developmental and mental disorders in younger individuals can have lasting effects that lead to significant social burden, special care and early intervention might be necessary [55, 56].

Although this study only examined changes during the pandemic period, it is necessary to further investigate how these changes persist after pandemic and how the prognosis of psychiatric disorders differs depending on the initial onset timing (before, during, and after the pandemic). In addition, when making policy decisions during future pandemics, it will be important to consider that public mental health may be influenced by either spread of infectious diseases or the societal responses related to the pandemic [2–5].

### Study limitations

This study has a few limitations that must be acknowledged. First, the diagnostic accuracy of psychiatric disorders in the health insurance data remains controversial. However, we estimated disease incidence changes over time, and the estimate is reliable as long as the diagnostic patterns for mental diseases remained the same before and during the pandemic [57]. South Korea maintained a strong medical care and health insurance system throughout the pandemic; therefore, diagnostic accuracy-related issues were not significantly different before and during the pandemic.

Second, instead of evaluating detailed disease subtypes, this study focused on major groups of psychiatric disorders based on the ICD-10 criteria. Additionally, vulnerability to psychiatric disorders in terms of COVID-19’s indirect effects was not addressed. Previous studies have shown that sex, socioeconomic status, and medical history can modify COVID-19’s influence on mental health [30]. However, detailed analysis of personal vulnerability (e.g., due to smoking, drinking, and body mass index) was not possible owing to data constraints. In addition, our study considered the country as a whole and did not assess different regional vulnerability to the impact of the pandemic on the development of psychiatric disorders. The purpose of this study was to evaluate the overall trend in the incidence of psychiatric disorders; therefore, the effect of individual and regional circumstances could be the focus of future studies.

Third, due to the descriptive nature of this study, even with the rigorous quasi-experimental design using interrupted time series analysis, the exact mechanisms behind the observed changes in psychiatric disorder incidence pattern cannot be pinpointed. Numerous factors affecting society emerged and disappeared during the pandemic, making it impossible to evaluate the effects of each factor. Therefore, we attempted to interpret the changes in disease incidence patterns during the pandemic by synthesizing available evidence from South Korea and other countries.

## Conclusion

To estimate the COVID-19 pandemic’s impact on the mental health of the population on a national scale, we evaluated the psychiatric disorder incidence patterns using nationwide health insurance data. During the pandemic, an increase in anxiety-related disorders, psychological developmental disorders, childhood and adolescent behavioral and emotional disorders, and mental retardation was observed. While the incidence rates of schizophrenia and mood disorders were stable, those of organic, including symptomatic, mental disorders and diseases related to psychoactive substance use decreased. The disruption of ordinary life and medical services may be the reason behind these changes. The causes and potential public health consequences of these changes should be monitored carefully.

## Supplementary Information

Supplementary Material 1.

## Data Availability

The data that support the findings of this study are available from National Health Insurance Service (https://nhiss.nhis.or.kr/bd/ay/bdaya001iv.do), Korea, but restrictions apply to the availability of these data, which were used under license for the current study, and so are not publicly available. Data are however available from the authors upon reasonable request and with permission of the National Health Insurance Service, Korea.
