# Supplementary material for: Changes in psychiatric disorder incidence patterns during the COVID-19 pandemic in Korea: a study using the nationwide universal health insurance data
Source: BMC Psychiatry. 2024 Dec 5;24:889. doi: 10.1186/s12888-024-06325-7 (PMC11619179; doi:10.1186/s12888-024-06325-7)
Supplement: Supplementary file 1 — Supplementary Material 1. [file 12888_2024_6325_MOESM1_ESM.docx]

Additional file 1

Table of contents

Table S1. The yearly population of Korea during the study period. Data provided by Statistics Korea

Table S2. The difference in observed and estimated age-standardized incidence rates; negative control analysis by setting random time point 2 years before the onset of COVID-19 pandemic in South Korea

Table S3. Sex-stratified estimation of the changes in monthly age-standardized disease incidence rates during the COVID-19 pandemic

Table S4. Changes in age-standardized disease-specific incidence rates by COVID-19 pandemic years

Table S5. Sensitivity analysis with pre-pandemic period limited to 2016.01–2020.02

Table S5. Sensitivity analysis with pre-pandemic period limited to 2017.01–2020.02

Figure S1. Observed and estimated age-standardized monthly disease incidence rates of psychiatric disease (F00 to F59) from January 2015 to February 2020. Middle dotted line represents estimated rates based on the pre-COVID19 pandemic disease incidence trend and solid line represents observed values. Upper and lower dotted lines represent 95% confidence intervals of the estimated rates.

Figure S2. Observed and estimated age-standardized monthly disease incidence rates of psychiatric disease (F60 to F99) from January 2015 to February 2020. Middle dotted line represents estimated rates based on the pre-COVID19 pandemic disease incidence trend and solid line represents observed values. Upper and lower dotted lines represent 95% confidence intervals of the estimated rates.

Table S1. The yearly population of Korea during the study period. Data provided by Statistics Korea

| Year | Total | Men | Women |
| --- | --- | --- | --- |
| 2013 | 51057575 | 25552389 | 25505186 |
| 2014 | 51242818 | 25632459 | 25610359 |
| 2015 | 51438644 | 25718466 | 25720178 |
| 2016 | 51623403 | 25797826 | 25825577 |
| 2017 | 51742229 | 25843482 | 25898746 |
| 2018 | 51803406 | 25861365 | 25942041 |
| 2019 | 51841928 | 25867238 | 25974690 |
| 2020 | 51840130 | 25853181 | 25986949 |
| 2021 | 51698023 | 25779374 | 25918649 |
| 2022 | 51547694 | 25696026 | 25851668 |
| 2023^a)^ | 51425748 | 25628874 | 25796874 |

^a)^ The mid-monthly populations for January and February 2023 were averaged to calculate the yearly population for 2023 to match the study period.

Table S2. The difference in observed and estimated age-standardized incidence rates; negative control analysis by setting random time point 2 years before the onset of COVID-19 pandemic in South Korea

| Disease categories (ICD-10 code) | Monthly disease  incidence rate (/1,000,000) : Observed | | Monthly disease  incidence rate (/1,000,000) : Estimated | | Changes in monthly   disease incidence rate^a)^ (/1,000,000) |
| --- | --- | --- | --- | --- | --- |
|  | 2015.01 -2018.02 | 2018.03 -2020.02 | 2015.01 -2018.02 | 2018.03 -2020.02 |  |
| Organic, including symptomatic, mental disorder (F00-F09) | 255.56 | 260.96 | 257.47 | 257.94 | 4.93 (-6.83, 16.69) |
| Mental and behavioral disorders due to psychoactive substance use (F10-F19) | 69.85 | 64.90 | 70.25 | 64.28 | 1.03 (-2.73, 4.78) |
| Schizophrenia, schizotypal and delusional disorders (F20-F29) | 38.59 | 36.58 | 38.84 | 36.18 | 0.65 (-1.43, 2.73) |
| Mood disorders (F30-F39) | 432.01 | 524.78 | 436.22 | 518.12 | 10.86 (-19.05, 40.78) |
| Neurotic, stress-related and somatoform disorders (F40-F48) | 656.87 | 674.50 | 658.84 | 671.37 | 5.10 (-35.04, 45.24) |
| Behavioral syndromes associated with physiological disturbances and physical factors (F50-F59) | 227.37 | 209.92 | 228.17 | 208.66 | 2.06 (-9.42, 13.53) |
| Disorders of adult personality and behavior (F60-F69) | 15.20 | 17.03 | 15.22 | 17.00 | 0.05 (-1.11, 1.21) |
| Mental retardation (F70-F79) | 33.21 | 29.08 | 33.17 | 29.14 | -0.10 (-2.12, 1.92) |
| Disorders of psychological development (F80-F89) | 45.72 | 49.06 | 46.24 | 48.24 | 1.33 (-2.23, 4.89) |
| Behavioral and emotional disorders with onset usually occurring in childhood and adolescence (F90-F98) | 108.77 | 126.40 | 109.75 | 124.85 | 2.53 (-8.47, 13.53) |
| Mental disorder NOS (F99) | 6.64 | 5.82 | 6.73 | 5.67 | 0.24 (-0.39, 0.87) |

^a)^ Comparison of observed and predicted counterfactual incidence rates for 2018.03–2020.02 period using difference-in-difference framework

Table S3. Sex-stratified estimation of the changes in monthly age-standardized disease incidence rates during the COVID-19 pandemic

| Disease (ICD-10 revision code) | Changes in incidence rate  during the pandemic^a)^ | | | Cochrane  Q statistics | p-value for  heterogeneity |
| --- | --- | --- | --- | --- | --- |
|  | Total | Men | Women |  |  |
| Organic, including symptomatic, mental disorder (F00-F09) | -37.78 (-47.52, -28.03)^b)^ | -27.22 (-35.4, -19.03)^b)^ | -47.5 (-58.96, -36.05)^b)^ | 9.17 | <0.01^b)^ |
| Mental and behavioral disorders due to psychoactive substance use (F10-F19) | -8.76 (-11.89, -5.63)^b)^ | -7.16 (-11.98, -2.33)^b)^ | -10.34 (-12.5, -8.17)^b)^ | 2.48 | 0.12 |
| Schizophrenia, schizotypal and delusional disorders (F20-F29) | 0.11 (-1.49, 1.71) | 0.42 (-1.21, 2.05) | -0.04 (-1.76, 1.68) | 0.17 | 0.68 |
| Mood disorders (F30-F39) | 22.42 (-12.49, 57.33) | -0.07 (-24.74, 24.61) | 45.13 (-1.71, 91.96) | 4.1 | 0.04^b)^ |
| Neurotic, stress-related and somatoform disorders (F40-F48) | 58.51 (20.39, 96.63)^b)^ | 29.99 (1.43, 58.56)^b)^ | 86.77 (37.61, 135.92)^b)^ | 5.1 | 0.02^b)^ |
| Behavioral syndromes associated with physiological disturbances and physical factors (F50-F59) | 17.82 (9.26, 26.38)^b)^ | 12.34 (4.94, 19.73)^b)^ | 23.96 (13.72, 34.21)^b)^ | 3.49 | 0.06 |
| Disorders of adult personality and behavior (F60-F69) | -0.43 (-1.41, 0.55) | 0.05 (-1.33, 1.44) | -0.87 (-1.53, -0.22)^b)^ | 2.17 | 0.14 |
| Mental retardation (F70-F79) | 2.76 (0.96, 4.56)^b)^ | 3.19 (0.96, 5.43)^b)^ | 2.28 (0.85, 3.71)^b)^ | 0.58 | 0.45 |
| Disorders of psychological development (F80-F89) | 9.21 (5.40, 13.01)^b)^ | 12.9 (7.48, 18.32)^b)^ | 5.28 (3.02, 7.55)^b)^ | 13.4 | <0.01^b)^ |
| Behavioral and emotional disorders with onset usually occurring in childhood and adolescence (F90-F98) | 52.28 (32.93, 71.64)^b)^ | 52.77 (29.37, 76.17)^b)^ | 51.97 (36.22, 67.71)^b)^ | 0 | 0.96 |
| Mental disorder NOS (F99) | 3.11 (2.39, 3.83)^b)^ | 3.15 (2.3, 4.01)^b)^ | 3.06 (2.42, 3.69)^b)^ | 0.03 | 0.86 |

^a)^ Comparison between observed and predicted counterfactual incidence rate

^b)^ p-value below 0.05

Table S4. Changes in age-standardized disease-specific incidence rates by COVID-19 pandemic years

| Disease categories  (ICD-10 code) | Monthly disease  incidence count (n) | Monthly disease  incidence rate (/1,000,000) | Changes in monthly   disease incidence rate (/1,000,000) compared to pre-pandemic | Percent change compared to pre-pandemic period (%) |
| --- | --- | --- | --- | --- |
| Organic, including symptomatic, mental disorder (F00-F09) |  |  |  |  |
| 2015.03-2020.02 | 23317 | 258 |  |  |
| 2020.03-2021.02 | 22718 | 219 | -38.56 (-55.89, -21.23)^a)^ | -14.9 (-21.7, -8.2)^a)^ |
| 2021.03-2022.02 | 24576 | 230 | -28.09 (-42.99, -13.19)^a)^ | -10.9 (-16.7, -5.1)^a)^ |
| 2022.03-2023.02 | 23599 | 211 | -46.68 (-56.9, -36.45)^a)^ | -18.1 (-22.1, -14.1)^a)^ |
| Mental and behavioral disorders due to psychoactive substance use (F10-F19) |  |  |  |  |
| 2015.03-2020.02 | 4317 | 68 |  |  |
| 2020.03-2021.02 | 3124 | 50 | -11.1 (-15.78, -6.42)^a)^ | -16.3 (-23.2, -9.4)^a)^ |
| 2021.03-2022.02 | 2970 | 48 | -10.21 (-14.52, -5.9)^a)^ | -15 (-21.4, -8.7)^a)^ |
| 2022.03-2023.02 | 3106 | 51 | -4.98 (-9.07, -0.89)^a)^ | -7.3 (-13.3, -1.3)^a)^ |
| Schizophrenia, schizotypal and delusional disorders (F20-F29) |  |  |  |  |
| 2015.03-2020.02 | 2358 | 38 |  |  |
| 2020.03-2021.02 | 2124 | 34 | -0.54 (-2.64, 1.56) | -1.4 (-6.9, 4.1) |
| 2021.03-2022.02 | 2084 | 34 | 0.46 (-1.96, 2.88) | 1.2 (-5.2, 7.6) |
| 2022.03-2023.02 | 1978 | 33 | 0.41 (-1.71, 2.53) | 1.1 (-4.5, 6.7) |
| Mood disorders (F30-F39) |  |  |  |  |
| 2015.03-2020.02 | 28619 | 468 |  |  |
| 2020.03-2021.02 | 31750 | 554 | -10.27 (-50.19, 29.66) | -2.2 (-10.7, 6.3) |
| 2021.03-2022.02 | 34306 | 628 | 33.57 (-2.55, 69.69) | 7.2 (-0.5, 14.9) |
| 2022.03-2023.02 | 35710 | 669 | 43.97 (-7.88, 95.82) | 9.4 (-1.7, 20.5) |
| Neurotic, stress-related and somatoform disorders (F40-F48) |  |  |  |  |
| 2015.03-2020.02 | 41984 | 664 |  |  |
| 2020.03-2021.02 | 40840 | 672 | -3.48 (-51.58, 44.61) | -0.5 (-7.8, 6.7) |
| 2021.03-2022.02 | 44734 | 765 | 85.57 (31.96, 139.17)^a)^ | 12.9 (4.8, 21.0)^a)^ |
| 2022.03-2023.02 | 44165 | 775 | 93.45 (33, 153.91)^a)^ | 14.1 (5.0, 23.2)^a)^ |
| Behavioral syndromes associated with physiological disturbances and physical factors (F50-F59) |  |  |  |  |
| 2015.03-2020.02 | 15599 | 221 |  |  |
| 2020.03-2021.02 | 14965 | 206 | 8.09 (-2.41, 18.6) | 3.7 (-1.1, 8.4) |
| 2021.03-2022.02 | 15274 | 210 | 19.49 (8.04, 30.93)^a)^ | 8.8 (3.6, 14.0)^a)^ |
| 2022.03-2023.02 | 15219 | 209 | 25.88 (15.42, 36.33)^a)^ | 11.7 (7.0, 16.4)^a)^ |
| Disorders of adult personality and behavior (F60-F69) |  |  |  |  |
| 2015.03-2020.02 | 729 | 16 |  |  |
| 2020.03-2021.02 | 764 | 17 | -0.82 (-1.98, 0.34) | -5.1 (-12.4, 2.1) |
| 2021.03-2022.02 | 791 | 18 | -0.23 (-1.79, 1.34) | -1.4 (-11.2, 8.4) |
| 2022.03-2023.02 | 816 | 19 | -0.26 (-1.56, 1.05) | -1.6 (-9.8, 6.6) |
| Mental retardation (F70-F79) |  |  |  |  |
| 2015.03-2020.02 | 1211 | 32 |  |  |
| 2020.03-2021.02 | 902 | 26 | -1.24 (-3.54, 1.06) | -3.9 (-11.1, 3.3) |
| 2021.03-2022.02 | 976 | 29 | 3.92 (1.58, 6.25)^a)^ | 12.2 (4.9, 19.5)^a)^ |
| 2022.03-2023.02 | 939 | 29 | 5.59 (3.56, 7.63)^a)^ | 17.5 (11.1, 23.8)^a)^ |
| Disorders of psychological development (F80-F89) |  |  |  |  |
| 2015.03-2020.02 | 1277 | 47 |  |  |
| 2020.03-2021.02 | 1254 | 55 | 5.01 (-2.01, 12.03) | 10.7 (-4.3, 25.6) |
| 2021.03-2022.02 | 1433 | 66 | 15.21 (10.06, 20.35)^a)^ | 32.4 (21.4, 43.3)^a)^ |
| 2022.03-2023.02 | 1264 | 59 | 7.4 (3.37, 11.43)^a)^ | 15.7 (7.2, 24.3)^a)^ |
| Behavioral and emotional disorders with onset usually occurring in childhood and adolescence (F90-F98) |  |  |  |  |
| 2015.03-2020.02 | 3494 | 116 |  |  |
| 2020.03-2021.02 | 4010 | 131 | -1.44 (-16.33, 13.45) | -1.2 (-14.1, 11.6) |
| 2021.03-2022.02 | 5642 | 190 | 51.49 (37.68, 65.29)^a)^ | 44.4 (32.5, 56.3)^a)^ |
| 2022.03-2023.02 | 7637 | 250 | 106.8 (86.79, 126.81)^a)^ | 92.1 (74.8, 109.3)^a)^ |
| Mental disorder NOS (F99) |  |  |  |  |
| 2015.03-2020.02 | 324 | 6 |  |  |
| 2020.03-2021.02 | 330 | 6 | 1.46 (0.53, 2.39)^a)^ | 24.3 (8.8, 39.8)^a)^ |
| 2021.03-2022.02 | 436 | 9 | 4.56 (3.65, 5.47)^a)^ | 76.0 (60.8, 91.2)^a)^ |
| 2022.03-2023.02 | 360 | 7 | 3.31 (2.34, 4.27)^a)^ | 55.2 (39.0, 71.2)^a)^ |

^a)^ p-value below 0.05

Table S5. Sensitivity analysis with pre-pandemic period limited to 2016.01–2020.02

| Disease categories (ICD-10 code) | Monthly disease  incidence count (n) | | Monthly disease  incidence rate (/1,000,000) | | Changes in monthly   disease incidence rate^c)^ (/1,000,000) | Percent change compared to prepandemic^d)^  (%) |
| --- | --- | --- | --- | --- | --- | --- |
|  | Before  pandemic^a)^ | During  pandemic^b)^ | Before  pandemic^a)^ | During  pandemic^b)^ |  |  |
| Organic, including symptomatic, mental disorder (F00-F09) | 23886 | 23631 | 258 | 220 | -35.09 (-45.23, -24.94)^e)^ | -13.6 (-17.5, -9.7)^e)^ |
| Mental and behavioral disorders due to psychoactive substance use (F10-F19) | 4201 | 3067 | 66 | 50 | -10.96 (-14.02, -7.9)^e)^ | -16.6 (-21.2, -12.0)^e)^ |
| Schizophrenia, schizotypal and delusional disorders (F20-F29) | 2317 | 2062 | 37 | 34 | -0.69 (-2.27, 0.89) | -1.9 (-6.1, 2.4) |
| Mood disorders (F30-F39) | 29063 | 33922 | 479 | 617 | 1.75 (-35.6, 39.11) | 0.4 (-7.4, 8.2) |
| Neurotic, stress-related and somatoform disorders (F40-F48) | 41848 | 43246 | 664 | 737 | 53.01 (12.06, 93.96)^e)^ | 8.0 (1.8, 14.2)^e)^ |
| Behavioral syndromes associated with physiological disturbances and physical factors (F50-F59) | 15408 | 15153 | 216 | 208 | 13.65 (5.46, 21.83)^e)^ | 6.3 (2.5, 10.1)^e)^ |
| Disorders of adult personality and behavior (F60-F69) | 740 | 790 | 16 | 18 | -0.56 (-1.6, 0.48) | -3.5 (-10, 3.0) |
| Mental retardation (F70-F79) | 1172 | 939 | 31 | 28 | 3.58 (1.72, 5.44)^e)^ | 11.5 (5.5, 17.5)^e)^ |
| Disorders of psychological development (F80-F89) | 1258 | 1317 | 47 | 60 | 5.91 (2.02, 9.8)^e)^ | 12.6 (4.3, 20.9)^e)^ |
| Behavioral and emotional disorders with onset usually occurring in childhood and adolescence (F90-F98) | 3551 | 5763 | 117 | 191 | 47.5 (27.62, 67.39)^e)^ | 40.6 (23.6, 57.6)^e)^ |
| Mental disorder NOS (F99) | 313 | 375 | 6 | 8 | 2.76 (2.05, 3.48)^e)^ | 46.0 (34.2, 58.0)^e)^ |

^a)^ Before pandemic (2015.01 - 2020.02)

^b)^ During pandemic (2020.03 - 2023.02)

^c)^ Comparison between observed and predicted counterfactual incidence rate

^d)^ Changes in monthly disease incidence rate/monthly disease incidence rate before pandemic

^e)^ p-value below 0.05

Table S6. Sensitivity analysis with pre-pandemic period limited to 2017.01–2020.02

| Disease categories (ICD-10 code) | Monthly disease  incidence count (n) | | Monthly disease  incidence rate (/1,000,000) | | Changes in monthly   disease incidence rate^c)^ (/1,000,000) | Percent change compared to prepandemic^d)^  (%) |
| --- | --- | --- | --- | --- | --- | --- |
|  | Before  pandemic^a)^ | During  pandemic^b)^ | Before  pandemic^a)^ | During  pandemic^b)^ |  |  |
| Organic, including symptomatic, mental disorder (F00-F09) | 24372 | 23631 | 257 | 220 | -38.7 (-49.84, -27.55)^e)^ | -15.1 (-19.4, -10.7)^e)^ |
| Mental and behavioral disorders due to psychoactive substance use (F10-F19) | 4116 | 3067 | 65 | 50 | -12.29 (-15.42, -9.16)^e)^ | -18.9 (-23.7, -14.1)^e)^ |
| Schizophrenia, schizotypal and delusional disorders (F20-F29) | 2289 | 2062 | 37 | 34 | -1.81 (-3.5, -0.12)^e)^ | -4.9 (-9.5, -0.3)^e)^ |
| Mood disorders (F30-F39) | 29620 | 33922 | 494 | 617 | -25.3 (-66.49, 15.88) | -5.1 (-13.5, 3.2) |
| Neurotic, stress-related and somatoform disorders (F40-F48) | 41555 | 43246 | 663 | 737 | 31.19 (-13.56, 75.93) | 4.7 (-2, 11.5) |
| Behavioral syndromes associated with physiological disturbances and physical factors (F50-F59) | 15249 | 15153 | 213 | 208 | 4.15 (-3.27, 11.57) | 1.9 (-1.5, 5.4) |
| Disorders of adult personality and behavior (F60-F69) | 751 | 790 | 16 | 18 | -1.14 (-2.27, 0) | -7.1 (-14.2, 0) |
| Mental retardation (F70-F79) | 1108 | 939 | 30 | 28 | 0.28 (-1.22, 1.79) | 0.9 (-4.1, 6) |
| Disorders of psychological development (F80-F89) | 1251 | 1317 | 48 | 60 | 3.27 (-0.89, 7.43) | 6.8 (-1.9, 15.5) |
| Behavioral and emotional disorders with onset usually occurring in childhood and adolescence (F90-F98) | 3619 | 5763 | 120 | 191 | 39.37 (18.67, 60.07)^e)^ | 32.8 (15.6, 50.1)^e)^ |
| Mental disorder NOS (F99) | 301 | 375 | 6 | 8 | 1.85 (1.13, 2.57)^e)^ | 30.8 (18.8, 42.8)^e)^ |

^a)^ Before pandemic (2015.01 - 2020.02)

^b)^ During pandemic (2020.03 - 2023.02)

^c)^ Comparison between observed and predicted counterfactual incidence rate

^d)^ Changes in monthly disease incidence rate/monthly disease incidence rate before pandemic

^e)^ p-value below 0.05





Figure S1. Observed and estimated age-standardized monthly disease incidence rates of psychiatric disease (F00 to F59) from January 2015 to February 2020. Middle dotted line represents estimated rates based on the pre-COVID19 pandemic disease incidence trend and solid line represents observed values. Upper and lower dotted lines represent 95% confidence intervals of the estimated rates.





Figure S2. Observed and estimated age-standardized monthly disease incidence rates of psychiatric disease (F60 to F99) from January 2015 to February 2020. Middle dotted line represents estimated rates based on the pre-COVID19 pandemic disease incidence trend and solid line represents observed values. Upper and lower dotted lines represent 95% confidence intervals of the estimated rates.
